# Controlled synthesis of two-dimensional porous molybdenum nitride *via* a stepwise nitridation growth mechanism

**DOI:** 10.1039/d5ra05246g

**Published:** 2025-10-24

**Authors:** Changbao Zhao, Jinjing Lu, Yibo Song, Lijun Xu, Wei Hu, Bin Wang

**Affiliations:** a Anhui Engineering Research Center of Highly Reactive Micro–Nano Powders, Chizhou University Chizhou 247000 China; b Feidu Aerospace Technology Co., Ltd Chizhou 247000 China huwei@fd-aerospace.com; c College of Chemistry and Materials Engineering, Bohai University Jinzhou 121013 China wangbinlhx@163.com

## Abstract

Transition metal nitrides exhibit desirable performance in various fields such as catalysis and energy storage due to their noble-metal-like properties. However, the controllable synthesis of two-dimensional (2D) planar metal nitrides with well-defined surfaces remains a challenge. Here, we report the controlled construction of molybdenum nitride model structures through stepwise nitridation using (NH_4_)_6_Mo_7_O_24_ as the precursor *via* chemical vapor deposition combined with a vapor–solid–solid (VSS) growth mechanism. Scanning electron microscopy (SEM), X-ray diffraction (XRD), and X-ray photoelectron spectroscopy (XPS) confirmed the successful fabrication of a well-ordered, 2D planar porous γ-Mo_2_N model structure on Al_2_O_3_(0001) substrates. By optimizing growth conditions, we achieved controllable synthesis of material morphology and structure. Raman spectroscopy analysis revealed that the prepared γ-Mo_2_N surface is rich in active sites, demonstrating significant interactions with adsorbed oxygen-containing small molecules, which confirms its excellent surface reactivity. This study provides a novel strategy for preparing model structures of transition metal nitrides and elucidates the critical role of the VSS mechanism in nitride thin film growth.

## Abstract 2

Transition metal nitrides exhibit desirable performance in various fields such as catalysis and energy storage due to their noble-metal-like properties.

## Graphical abstract

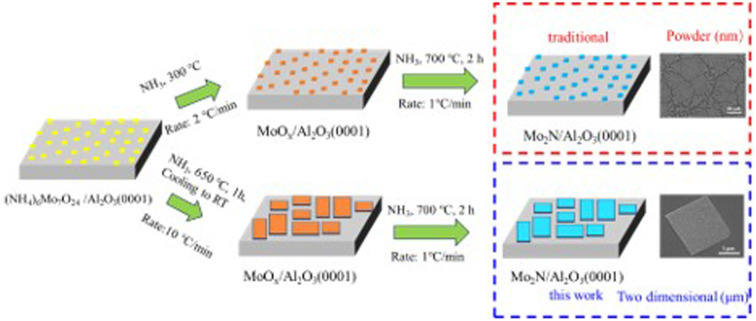

## Introduction

Transition metal carbides and nitrides (TMC_*x*_, TMN_*x*_), as emerging members of the 2D material family, originate from parent ceramic materials with ternary layered structures. The uniqueness of these materials lies in the transfer of outer electrons from carbon (C) or nitrogen (N) to the d-orbitals of the transition metals, resulting in an outer electron orbital configuration of the metals that resembles that of noble metal platinum (Pt).^1^ This significant discovery has spurred in-depth research into other early transition metal carbides and nitrides. Owing to their exceptional physicochemical properties, TMC_*x*_ and TMN_*x*_ exhibit broad application prospects in catalytic fields such as hydrocarbon isomerization, hydrogenation reactions, and electrochemical hydrogen evolution reactions.^2–6^ Studies indicate that factors such as crystal structure, site occupancy, metal-to-carbon/nitrogen ratio, surface termination, and defects can significantly influence the catalytic activity of TMC_*x*_ and TMN_*x*_.^7,8^ In addition to being directly used as a catalyst, TMC_*x*_ and TMN_*x*_ are also employed as a catalyst support for loading active metals.^9–12^ The strong interaction between the metal and the TMN_*x*_ support enables the active metal to achieve a highly dispersed state on the TMN_*x*_ surface, along with exceptional anti-sintering properties.^13,14^ In summary, whether as a catalyst or a support, molybdenum nitride represents a highly promising new catalytic material that warrants in-depth investigation and further research.

However, the elementary reaction steps and active structures of TMN_*x*_ during catalytic processes still require in-depth understanding. For example, Zhang *et al.* discovered that in the water–gas shift reaction, the molybdenum species on the surface of Pt/γ-Mo_2_N catalysts can be readily reduced and transformed into MoO_*x*_ species, significantly enhancing the ability to dissociate H_2_O molecules.^15^ Furthermore, Xin *et al.* observed that during CO_2_ hydrogenation, the surface of Mo_2_N catalysts undergoes carburization to form MoC_*x*_ structures, which strengthens the adsorption and activation of CO_2_/H_2_, thereby further promoting CO_2_ hydrogenation conversion.^16^ Therefore, gaining fundamental insights into the structural evolution and surface chemistry of TMN_*x*_ at the atomic level is particularly crucial for optimizing their catalytic performance. The inherent diversity and complexity of variables in practical catalytic systems pose significant challenges in identifying their catalytically active configurations. To reduce system complexity, well-defined model catalysts with controlled surface structures serve as powerful tools for precisely identifying active sites and reaction intermediates.^17,18^ This necessitates the controlled synthesis of ultrathin, epitaxial, and well-defined TMN_*x*_ model catalysts for mechanistic studies.

Herein, we report the controlled synthesis of porous 2D molybdenum nitride (γ-Mo_2_N) *via* chemical vapor deposition (CVD) employing a vapor–solid–solid (VSS) growth mechanism, using (NH_4_)_6_Mo_7_O_24_ as the precursor. Comprehensive characterization techniques including scanning electron microscopy (SEM), X-ray diffraction (XRD), and X-ray photoelectron spectroscopy (XPS) confirmed the successful fabrication of well-defined, planar γ-Mo_2_N nanostructures with porous architecture on Al_2_O_3_(0001) substrates. By systematically optimizing growth parameters, we achieved precise control over the material's morphology and structure. Raman spectroscopy analysis revealed abundant active sites on the γ-Mo_2_N surface that exhibit strong interactions with adsorbed oxygen-containing molecules (*e.g.*, H_2_O, O_2_), demonstrating exceptional surface reactivity. This study not only provides a novel strategy for fabricating model transition metal nitride structures but also elucidates the crucial role of VSS mechanisms in nitride thin film growth.

## Experiments

### Substrate preparation

The Al_2_O_3_(0001) substrate surfaces underwent multiple cleaning cycles using ethanol followed by deionized water. After thorough rinsing with copious amounts of deionized water, the substrates were dried using nitrogen gas. To enhance their hydrophilic properties, select substrates received additional UV-ozone treatment for 30 minutes.

### Catalyst preparation

To prepare the Mo_2_N model catalysts, a 0.2 mL aliquot of Na_2_MoO_4_ aqueous solution (500 mg mL^−1^) was spin-coated onto the Al_2_O_3_(0001) substrate at 5000 rpm.

Conventional Temperature-Programmed Nitridation (TPN) Method: the Mo_2_N catalysts were synthesized *via* a conventional temperature-programmed nitridation approach. The precursor was heated in flowing NH_3_ atmosphere with a temperature ramp of 2 °C min^−1^ up to 300 °C, followed by a reduced heating rate of 1 °C min^−1^ to reach 700 °C. The sample was then maintained at this temperature for 2 h under NH_3_ flow before being cooled to room temperature.

Stepwise Nitridation Method: firstly, the precursor was heated in Ar atmosphere at 10 °C min^−1^ to 650 °C. Upon reaching the target temperature, the gas was switched to NH_3_ and held for 1 hour before cooling to room temperature. Then, the intermediate product subsequently underwent temperature-programmed nitridation in NH_3_ atmosphere with a heating rate of 1 °C min^−1^ to 700 °C, followed by a 2 h dwell at this final temperature before cooling to room temperature.

## Results and discussion

**Fig. 1:**
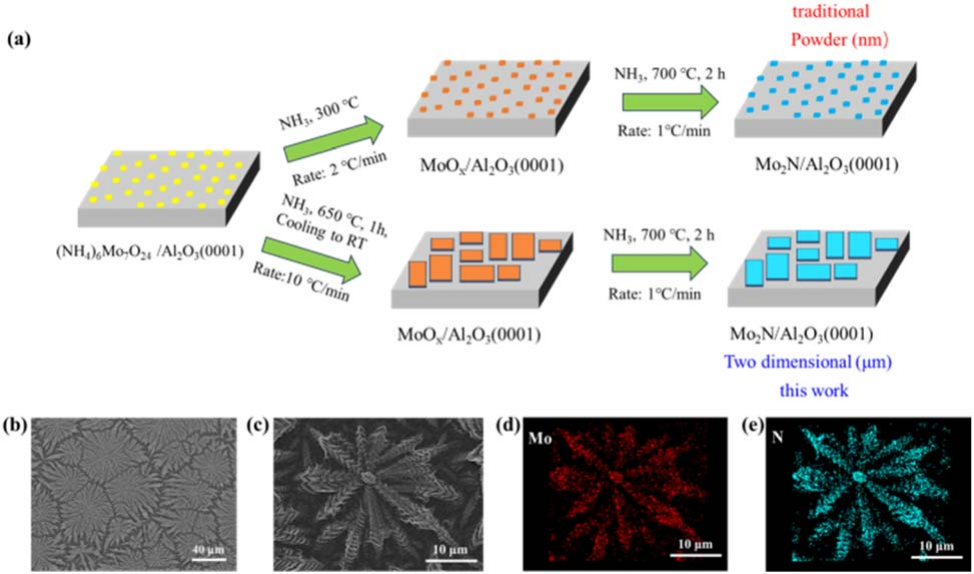
(a) Schematic illustration of the VSS growth process of Mo_2_N. (b and c) Typical SEM images of the Mo_2_N crystals grown with traditional VSS mode. (d and e) EDX elemental mappings of N and Mo of the Mo_2_N.

An ambient-pressure CVD approach was utilized to synthesize ultrathin Mo_2_N overlayers on Al_2_O_3_(0001) surfaces. The growth scheme *via* VSS is illustrated in Fig. 1a. Traditional methods for Mo_2_N growth primarily involve the thermal decomposition of (NH_4_)_6_Mo_7_O_24_ precursors followed by nitridation in an ammonia atmosphere (the upper panel in Fig. 1a). However, such conventional approaches typically yield irregular Mo_2_N powder structures, making it challenging to achieve controlled growth of 2D planar architectures.^16^ It is noteworthy that well-structured 2D porous Mo_2_N micron sheets were successfully fabricated *via* a VSS growth mechanism combined with the stepwise nitridation method, as schematically illustrated in the bottom panel of Fig. 1a. The schematic clearly reveals uniformly distributed, well-aligned rectangular structures on the substrate surface.

As shown in Fig. 1b and c, SEM characterization reveals that Mo_2_N synthesized *via* traditional methods does not form well-defined 2D morphologies but instead exhibits typical fractal growth features. Energy-dispersive X-ray spectroscopy (EDS) elemental mapping confirms that despite the fractal morphology, the Mo and N elements maintain a uniform spatial distribution within the microsheets, demonstrating the homogeneity of the nitridation process (Fig. 1d and e). The formation of such fractal structures may be attributed to the random distribution of nucleation sites and anisotropic growth kinetics during vapor-phase deposition. These observations highlight the inherent limitations of conventional CVD processes in controlling 2D morphology.^19^

**Fig. 2:**
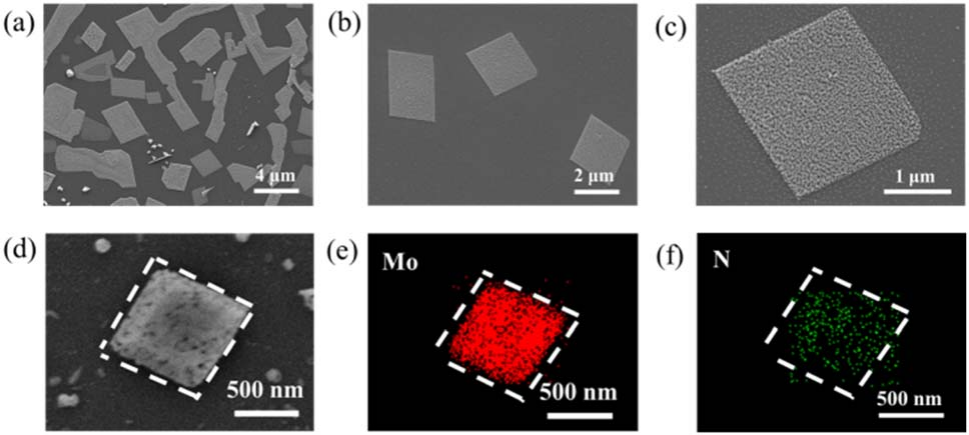
(a–d) Typical SEM images of the Mo_2_N crystals grown with stepwise nitridation VSS mode. (e and f) Energy dispersive X-ray (EDX) elemental mappings of N and Mo of the Mo_2_N.

Fig. 2 presents the microstructural morphology and elemental distribution characteristics of molybdenum nitride prepared in this study through a vapor–solid–solid (VSS) growth mechanism combined with a stepwise ammoniation strategy. As shown in Fig. 2a, well-defined 2D layered structures with regular geometric morphologies, primarily rectangular and elongated shapes were successfully grown on the Al_2_O_3_(0001) substrate, demonstrating the remarkable anisotropic control of crystal growth by the VSS mechanism. Notably, due to inevitable thickness variations during precursor spin-coating, localized agglomerates persist in certain regions. Further statistical analysis of the morphology (Fig. 2b) reveals that the obtained Mo_2_N microsheets exhibit an average lateral size of 2–3 μm, though some structures exceed 20 μm.

In contrast to conventional dense architectures, high-magnification SEM images (Fig. 2c) clearly reveal a unique loose and porous morphology. This open three-dimensional pore structure likely originates from the stage-specific regulation of reaction kinetics during stepwise ammoniation. To verify chemical homogeneity, we performed EDS mapping on representative microsheets (Fig. 2d–f). The results confirm that Mo and N elements maintain highly uniform spatial distribution within the porous framework, highlighting the superiority of the stepwise nitridation VSS mechanism in preserving stoichiometric ratios. Furthermore, the specific surface area of Mo_2_N was determined to be 119.1 m^2^ g^−1^, consistent with literature reports.^17^ The synergistic combination of this porous structure and homogeneous elemental distribution may endow Mo_2_N with distinctive structural advantages for catalytic or energy storage applications.

Critical experimental parameters, including growth temperature, reaction time, and ammonia flow rate significantly influence the morphological evolution of Mo_2_N. A higher reaction temperature (*e.g.*, 700 °C) facilitates precursor decomposition and atomic diffusion, promoting the formation of a 2D porous nanosheet structure and grain growth. Prolonged reaction time aids in completing the stepwise nitridation and avoiding the retention of intermediate phases, thereby yielding structurally intact γ-Mo_2_N. The ammonia flow rate directly governs the supply of active nitrogen species. A higher flow rate accelerates the nitridation reaction and promotes the growth of a porous bulk structure, whereas an appropriately lower flow rate facilitates the formation of layered γ-Mo_2_N. These parameters collectively regulate the phase transformation pathway from MoO_3_ to γ-Mo_2_N and the interfacial reaction kinetics, ultimately enabling the controlled fabrication of 2D porous Mo_2_N.

**Fig. 3:**
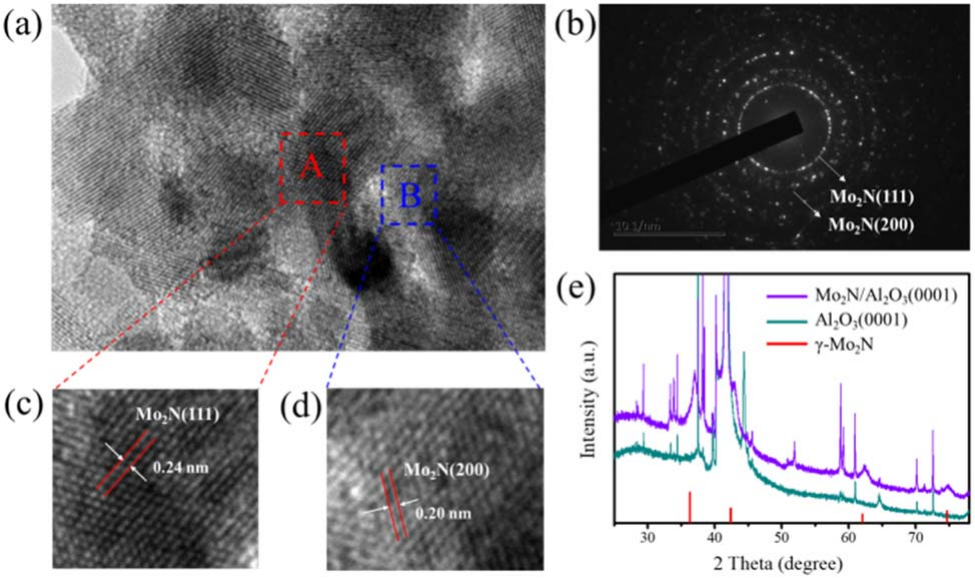
(a) HRTEM image of Mo_2_N. (b) SAED pattern of Mo_2_N. (c) The lattice fringes of Mo_2_N in region A of Fig. 4a. (d) The lattice fringes of Mo_2_N in region B of Fig. 4a. (e) XRD pattern of the Mo_2_N/Al_2_O_3_(0001) surface.

High-resolution transmission electron microscopy (HRTEM) was employed for systematic microstructural characterization of molybdenum nitride. As shown in Fig. 3a, the HRTEM image clearly reveals that the 2D layered structure consists of numerous randomly oriented and stacked nanocrystals with uniform size distribution and well-defined grain boundaries. Lattice fringe analysis indicates significant orientation differences between adjacent grains, forming a typical polycrystalline interface structure, confirming that the synthesized molybdenum nitride is a polycrystalline 2D material.^20^ Further selected-area electron diffraction (SAED) analysis (Fig. 3b) demonstrates that the diffraction pattern exhibits characteristic concentric rings composed of multiple diffraction spots.^21^ This diffraction feature originates from the collective contribution of a large number of randomly oriented nanocrystals within the material, with each diffraction ring corresponding to Bragg diffraction from different crystal planes. These results corroborate the polycrystalline structure observed in HRTEM.

To further elucidate the crystallographic characteristics of the material, high-resolution lattice analysis was conducted on regions A and B marked in Fig. 3a. As shown in Fig. 3c, the HRTEM result reveals distinct lattice fringes with a spacing of 0.24 nm in region A, which perfectly matches the (111) interplanar distance of γ-Mo_2_N. In contrast, region B (Fig. 3d) exhibits a lattice spacing of 0.20 nm, corresponding to the (200) plane of γ-Mo_2_N. Notably, the two adjacent regions display different crystallographic orientations, and no definitive epitaxial relationship with the substrate is observed. This phenomenon confirms that the material adopts a polycrystalline structure grown in a non-epitaxial manner.

The crystal structure and phase composition of the molybdenum nitride catalyst were systematically analyzed by XRD. As shown in Fig. 3e, the XRD pattern of the sample exhibits a series of characteristic diffraction peaks, the positions and relative intensities of which perfectly match the standard diffraction card of γ-Mo_2_N (PDF#25-1366). Specifically, the characteristic diffraction peaks observed at 2*θ* = 37.4°, 43.5°, 63.1°, and 75.7° correspond to the (111), (200), (220), and (311) planes of the cubic γ-Mo_2_N phase, respectively.^22^ The sharp profiles and high intensities of these diffraction peaks indicate excellent crystallinity of the synthesized molybdenum nitride sample. Additionally, all other diffraction peaks in the XRD pattern can be attributed to signals from the Al_2_O_3_(0001) substrate, with no impurity peaks detected. This confirms the high phase purity of the sample and the absence of any secondary phases. These results are consistent with the observations from HRTEM image, further verifying that the synthesized γ-Mo_2_N sample possesses a single-phase composition and high crystalline quality.

**Fig. 4:**
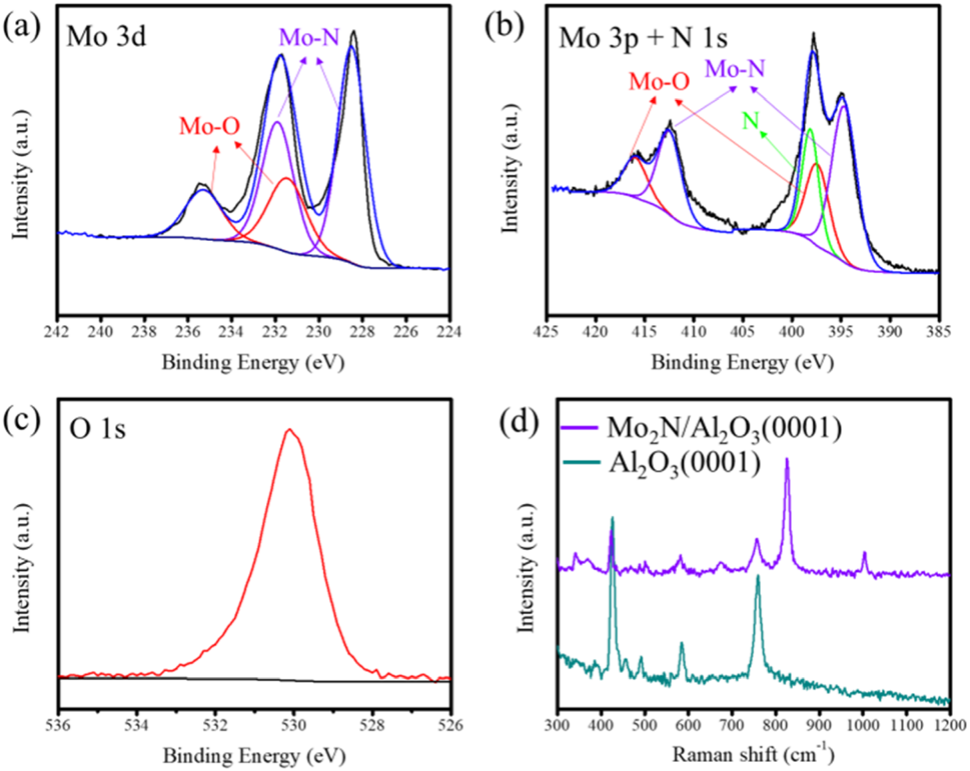
(a–c) XPS spectra acquired at the Mo 3d, Mo 3p + N 1s and O 1s regions. (d) Raman spectra of the Mo_2_N/Al_2_O_3_(0001) surface.

To investigate the chemical state and stoichiometric ratio of molybdenum nitride in depth, the sample was systematically characterized by XPS. Fig. 4a shows the high-resolution XPS spectrum of Mo 3d, which can be deconvoluted into four characteristic peaks after peak fitting. Among them, the doublet peaks located at binding energies of 228.6 eV and 231.7 eV correspond to Mo 3d_5/2_ and Mo 3d_3/2_, respectively, and are attributed to the low-valence Mo^*δ*+^ (0 < *δ* < 2), indicating the formation of Mo–N bonds.^23,24^ In contrast, the doublet peaks at 232.1 eV and 235.2 eV are assigned to Mo^6+^ (Mo 3d_5/2_ and Mo 3d_3/2_), suggesting partial surface oxidation (Mo–O).^25^ This oxidation likely originates from a thin layer of MoO_*x*_ formed on the sample surface upon exposure to air, though Mo–N remains the predominant component.

Furthermore, due to the overlapping binding energy ranges of Mo 3p_3/2_ (∼394.6 eV) and N 1s (∼397.8 eV), peak deconvolution was performed to further analyze this region. The fitting results revealed that the peaks at 394.6 eV and 412.6 eV correspond to Mo 3p_3/2_ and Mo 3p_1/2_, respectively, which are also attributed to the Mo^*δ*+^–N bonds, further confirming the presence of Mo–N bonding (Fig. 4b). Meanwhile, the peaks at 397.3 eV and 416.0 eV are assigned to Mo^6+^–O bonds, consistent with the analysis of the Mo 3d spectrum. The characteristic peak at 397.8 eV is attributed to N 1s, indicating that nitrogen exists in a chemically bonded form (Mo–N).^26,27^ Quantitative analysis of the peak areas of Mo 3d and N 1s revealed an atomic ratio of Mo to N of approximately 2 : 1. It should be noted that, although every effort was made to minimize air exposure during sample transfer, slight surface oxidation remains practically unavoidable. This may lead to an increased contribution from the Mo–O peak, thereby affecting the accuracy of atomic ratio calculations such as Mo/N. Nevertheless, after deducting the proportion of high-valence oxidized molybdenum and applying standard sensitivity factors to the nitrogen signal, the semi-quantitative Mo/N atomic ratio obtained can be considered reasonably accurate. Additional, combined with XRD and TEM results, this confirms that the synthesized sample is a molybdenum nitride material with a stoichiometry close to Mo_2_N.

It is worth noting that the analysis of XPS spectra, including Mo 3d, Mo 3p, and O 1s (Fig. 4c), consistently revealed the presence of Mo–O bonding. This observation indicates that the surface of Mo_2_N possesses high reactivity in air, readily interacting with oxygen-containing small molecules and forming a surface oxide layer through oxidation. To further confirm the formation of this surface oxide layer, Raman spectroscopy was employed to characterize the Mo_2_N surface. As shown in Fig. 4d, Raman spectroscopy analysis reveals distinct characteristic peaks at 820 cm^−1^ and 996 cm^−1^, which are attributed to the stretching vibration of Mo–O–Mo bridge bonds and the symmetric stretching of terminal Mo=O bonds in MoO_3_, respectively. The precise position of the peak at 996 cm^−1^ indicates the presence of highly oxidized hexagonal-phase MoO_3_, consistent with reference spectra.^28^ These results further demonstrate that the as-synthesized γ-Mo_2_N features abundant active sites on its surface, enabling strong interactions with adsorbed oxygen-containing molecules and exhibiting exceptional surface reactivity.^15^

The above results confirm that using (NH_4_)_6_Mo_7_O_24_ as a precursor enables the fabrication of 2D porous Mo_2_N microsheets with a well-defined surface morphology. We propose that the growth mechanism involves the initial decomposition of (NH_4_)_6_Mo_7_O_24_ into MoO_3_, followed by the removal of oxygen from MoO_3_ by hydrogen generated from the dissociation of NH_3_ at high temperatures, ultimately leading to the reaction between N and Mo to form Mo_2_N. In this study, rapid heating in ammonia was first employed because the degree of nitridation at this stage is relatively low, which promotes the sublimation of excess MoO_3_ below its melting point. This effectively restricts the thickness of the precursor. Additionally, the wettability of the Al_2_O_3_(0001) substrate surface and its interaction with MoO_3_ facilitate 2D layered growth. The Al_2_O_3_(0001) substrate possesses a hexagonal atomic symmetry. A commensurability exists with the γ-Mo_2_N crystal structure, which, while face-centered cubic, presents a hexagonal arrangement on its (111) plane. This lattice matching facilitates the establishment of a low interfacial energy between the Mo_2_N (111) plane and the Al_2_O_3_(0001) surface. From a thermodynamic perspective, the reduced interfacial energy markedly diminishes the initial nucleation barrier. This promotes an epitaxial or quasi-epitaxial growth mode, where Mo_2_N nuclei spread laterally across the substrate, as opposed to the Volmer–Weber mode characterized by three-dimensional island formation.

Furthermore, at the initial nitridation temperature, intermediate products (*e.g.*, MoO_3_) and sub-stoichiometric oxides (MoO_3−*x*_) exhibit high volatility. Selective sublimation of molybdenum oxide molecules from the solid surface or interior leaves behind voids. This volatilization process intensifies mass loss at the reaction front and, acting synergistically with vacancy formation, accelerates pore widening and interconnection, ultimately leading to an interpenetrating porous network. Simultaneously, adjacent γ-Mo_2_N nanocrystals with similar crystallographic orientations rotate, migrate, and align along specific crystal planes under interfacial forces, directly connecting to form larger single-crystalline domains.

Subsequent stepwise slow nitridation was implemented because the nitridation process of molybdenum involves multiple reaction steps, potentially generating various intermediate phases (MoO_3_ → MoO_2_ → Mo_3_N_2_ → Mo_2_N). If the heating or nitridation rate is too fast, incomplete nitridation may occur, leaving residual MoO_2_ or low-valence nitrides (*e.g.*, Mo_3_N_2_), thereby compromising the purity of the final product. Stepwise heating allows for the gradual removal of oxygen and promotes uniform nitridation, ensuring the formation of single-phase γ-Mo_2_N.

Therefore, we conclude that the Al_2_O_3_(0001) substrate induces the epitaxial growth of MoO_3_ with exposed low-energy crystal planes. Moreover, during the solid–solid interfacial reaction between active nitrogen species generated from NH_3_ cracking and molybdenum oxides, the difference in surface atomic coordination number across various crystal planes leads to anisotropic nitridation kinetics. The inheritance of crystallographic orientation from intermediate phases further constrains the final crystal orientation of the product. In summary, the VSS mechanism achieves cross-scale control from atomic-level gas–solid interfacial reactions to microscopic crystal orientation through interfacial energy regulation and diffusion kinetic constraints. This strategy provides a physicochemical design principle for the oriented preparation of 2D nitride materials.

**Fig. 5:**
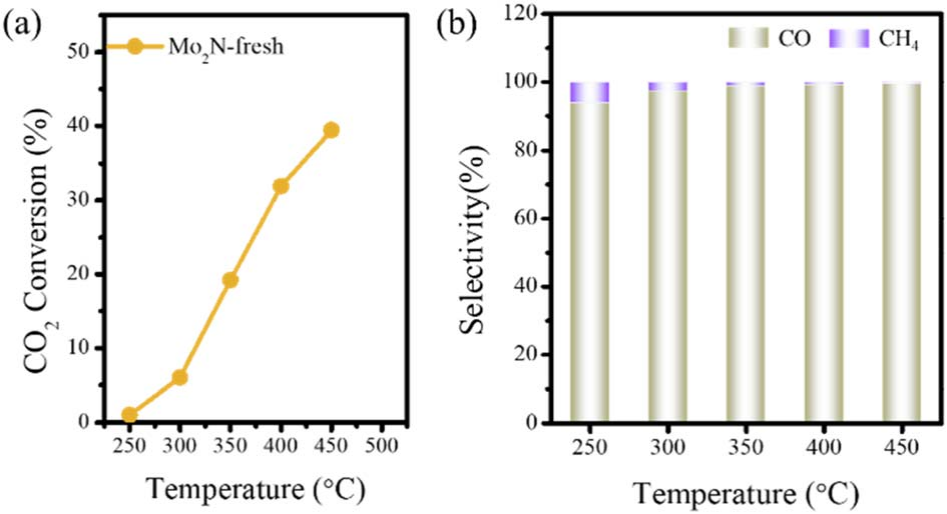
(a) CO_2_ conversion on the fresh Mo_2_N catalysts under reaction condition of 50 mg catalysts. (b) CO and CH_4_ selectivity on the fresh Mo_2_N catalysts under reaction condition of 50 mg catalysts.

Additionally, an evaluation of the CO_2_ hydrogenation reaction performance was carried out on the obtained 2D porous Mo_2_N. As illustrated in Fig. 5a, as the reaction temperature increases from 250 °C to 450 °C, the CO_2_ conversion rate of the 2D porous Mo_2_N catalyst rises from 1% to 39.5%. Notably, the selectivity of the products exhibits dynamic variations with increasing temperature. When the reaction temperature is below 350 °C, a small amount of CH_4_ is generated (Fig. 5b). At temperatures above 350 °C, CO becomes the dominant product with a selectivity as high as 99%, indicating that the Mo_2_N catalyst primarily exhibits reverse water–gas shift reaction performance in the CO_2_ hydrogenation reaction.

## Conclusions

Well-defined 2D porous γ-Mo_2_N microsheets with a high specific surface area can be controllably synthesized on Al_2_O_3_(0001) substrates *via* a stepwise nitridation strategy, using (NH_4_)_6_Mo_7_O_24_ as the precursor in a CVD process coupled with a VSS growth mechanism. Ammonia-assisted stepwise nitridation drives morphological reconstruction of Mo_2_N, leading to the formation of planar 2D structures. Furthermore, the as-prepared Mo_2_N demonstrates highly reactive surface properties, enabling its application as an active catalyst for adsorbing oxygen-containing small molecules. This work not only provides an effective strategy for fabricating TMN_*x*_ model structures but also establishes a fundamental basis for future research and applications of nitride thin-film materials.

## Author contributions

Wei Hu and Bin Wang designed the experiment and analyzed the data. Changbao Zhao conducted the VSS growth and analyzed the data. All the authors discussed and commented on the manuscript.

## Conflicts of interest

The authors declare that they have no known competing financial interests or personal relationships that could have appeared to influence the work reported in this paper.

## Data Availability

The data sets used and analyzed during the current study are available from the corresponding author on reasonable request.
